# Nemo-Like Kinase Associated with Proliferation and Apoptosis by c-Myb Degradation in Breast Cancer

**DOI:** 10.1371/journal.pone.0069148

**Published:** 2013-07-23

**Authors:** Yeqing Huang, Ying Jiang, Weiqi Lu, Yong Zhang

**Affiliations:** 1 Department of Tumor Chemotherapy, Affiliated Hospital of Nantong University, Medical College, Nantong University, Nantong, Jiangsu, China; 2 Department of General Surgery, Zhongshan Hospital, Fudan University, Shanghai, China; University of Medicine and Dentistry of New Jersey, United States of America

## Abstract

Nemo-like kinase (NLK), a mediator of the Wnt signaling pathway, binds directly to c-Myb, leading to its phosphorylation, ubiquitination and proteasome-dependent degradation. NLK was significantly downregulated in the breast cancer tissues compared to corresponding normal tissues. NLK expression was negatively correlated with c-Myb expression. NLK suppressed proliferation, induced apoptosis and mediated c-Myb degradation in MCF-7 cells via a mechanism that seems to involve c-myc and Bcl2. These findings might provide a novel target for therapeutic intervention in patients with breast cancer.

## Introduction

Breast cancer is one of the most debilitating human carcinomas and has the second highest mortality rate after lung cancer in women [1]. Although there is a very large body of information on the development and progression of breast cancer, all key factors have not yet been elucidated.

Wnt proteins belong to a large family of secreted signaling molecules that direct cell growth and fate [2]. Several lines of recent evidence show that the Wnt pathway is critical for the development of a normal mammary gland, whereas aberrant Wnt signaling is observed in cancer [1]. The Nemo-like kinase (NLK) of the Wnt signaling pathway is a member of the extracellular-signal regulated kinase/microtubule-associated protein kinase (Erk/MAPK) and cyclin-dependent kinase (Cdk) families [3]. NLK functions downstream of transforming growth factor β-activated kinase1 (TAK1), which is a member of the mitogen-activated protein kinase kinase kinase (MAPKKK or MAP3K) family [4]. NLK is a multifaceted cell signaling regulator [5]. NLK has been shown to be homologous to the Drosophila nemo gene [6], which is important for head [7] and wing development in Drosophila, as well as cell division in C. elegans [8], [9]. NLK induces apoptosis and inhibits AR-mediated transcriptional activity in prostate cancer cells [10]; however, it also contributes to tumor cell growth through the activation of the cell cycle transition in human hepatocellular carcinoma [3]. NLK induces apoptosis in glioma cells via activation of caspases [11]. Thus, NLK has been shown to be a critical regulator of cell growth, development and death in a variety of organisms.

c-Myb is a DNA-binding transcription factor that regulates the expression of specific genes during cell development and differentiation in various cell types [12], [13]. c-Myb has been involved in the regulation of hematopoietic [14]–[16], colon, mammary and endothelial [17]–[21] cell proliferation. MYB expression correlates with poor clinical prognosis in colon tumors [22], and an important transcriptional regulatory region of MYB is frequently mutated in this disease [23], [24]. Furthermore, MYB is required for colon carcinoma cell proliferation and is downregulated during the differentiation of these cells [25], [26], [23]. Most importantly, MYB suppresses the differentiation and apoptosis of human breast cancer cells [27].

Recent studies have indicated that c-Myb is phosphorylated and degraded via the Wnt-1 signaling pathway involving TGF-β-activated kinase1 (TAK1), homeodomain-interacting protein kinase 2 (HIPKs) and NLK [28]. NLK binds directly to c-Myb, leading to its phosphorylation, ubiquitination and proteasome-dependent degradation [29]. Wnt signaling was reported to have an important role in the growth regulation of mammary epithelial cells [30], [31]. Therefore, Wnt-dependent downregulation of c-Myb activity may play a critical role in controlling the proliferation and differentiation of mammary epithelial cells [28].

In this study, we used immunohistochemical analysis to determine whether there is a strong negative association between NLK and cytoplasmic c-Myb in breast carcinoma specimens, and we compared those findings with clinical outcomes. We transfected MCF-7 cells with an NLK expression vector and found that c-Myb levels were substantially reduced. These results link NLK to c-Myb and outline a regulatory pathway that is likely to affect the proliferation and apoptosis of breast cancer cells. The role of this regulatory pathway in breast cancer therapy was assessed.

## Materials and Methods

### Tissue Samples

Breast cancer specimens (n = 62) were obtained from patients who underwent surgery between 2005 and 2009 at the Department of General Surgery, Affiliated Hospital of Nantong University. The samples were formalin-fixed and paraffin-embedded for histopathologic diagnosis and immunohistochemical analysis. Fresh samples were frozen in liquid nitrogen immediately after surgical removal and maintained at −80°C until used for Western blotting. All human tissue samples were collected using protocols approved by the Ethics Committee of Affiliated Cancer Hospital of Nantong University. All the patients provided their written informed consent to participate in this study. It is approved by the ethics committees of Affiliated Cancer Hospital of Nantong University. The clinical features of the patients, including age, histologic grade, tumor size, metastasis, histology, as well as ER, PR and Her2 status, are shown in Table 1. The median age of the patients was 47 years (range: 27–79 years). Histologic grades were defined as well (grade I; n = 16), moderately differentiated (grade II; n = 23), and poorly differentiated (grade III; n = 23). The majority of tumors (n = 48, 77.4%) were infiltrating ductal carcinomas, and the remaining 14 cases were of other types. Details are shown in Table 1.

**Table 1:** NLK c-Myb expression and clinicopathological parameters in 62 breast cancer specimens.

Parameters	total	NLK		P	c-Myb		P
		Low ≦0.19	High >0.19		Low ≦3	High >3	
Age(years)							
≤47	40	26	14	0.533	12	28	0.058
>47	22	16	6		12	10	
Histological grade							
Well	16	2	14	<0.001*	10	6	0.064
Mod	23	19	4		8	15	
Poor	23	21	2		6	17	
Metastasis							
Positive	35	22	13	0.349	15	20	0.445
Negative	27	20	7		9	18	
Tumor size (cm)							
≤5	43	30	13	0.608	15	28	0.882
>5	19	12	7		7	12	
Histology							
Ductal	48	33	15	0.753	18	30	0.717
Others	14	9	5		6	8	
ER							
+	34	26	8	0.105	14	20	0.66
−	28	16	12		10	18	
PR							
+	34	26	8	0.105	14	20	0.66
−	28	16	12		10	18	
Her2							
+	37	29	8	0.029*	16	21	0.373
−	25	13	12		8	17	
Ki67							
≤0.60	29	11	18	<0.001*	18	11	<0.001*
>0.60	33	31	2		6	27	

Statistical analyses were performed by the Pearson χ2 test.

* P<0.05 is considered significant.

### Immunohistochemical Methods

Sections were deparaffinized using a graded ethanol series, and endogenous peroxidase activity was blocked with 0.3% hydrogen peroxide. Subsequently, the sections were processed in 10 mmol/L citrate buffer (pH 6.0) and heated to 121°C in an autoclave machine for 20 minutes to retrieve the antigen. After rinsing with PBS (pH 7.2), the sections were blocked with 10% goat serum for 1 hour at room temperature. The sections were then incubated overnight at 4°C with rabbit anti-human NLK polyclonal antibody (diluted 1∶100; Santa Cruz Biotechnology), rabbit anti-human c-Myb polyclonal antibody (diluted 1∶100; Santa Cruz Biotechnology) and mouse anti-Ki-67 monoclonal antibody (diluted 1∶100; clone 7B11; Zymed Laboratories, San Francisco, Calif., USA). Negative control slides were processed in parallel using a nonspecific immunoglobulin IgG (Sigma Chemical Co., St. Louis, MO) at the same concentration as the primary antibody. All the slides were processed using the peroxidase-antiperoxidase method (Dako, Hamburg, Germany). After rinsing the sections with PBS, the peroxidase reaction product was visualized by incubating the sections with diaminobenzidine tetrahydrochloride in 0.05 mol/L Tris buffer (pH 7.6) containing 0.03% H2O2. The sections were then rinsed with water, counterstained with hematoxylin, dehydrated, and mounted. All the immunostained sections were evaluated by observers blinded to the clinical and pathological parameters of the patients.

### Evaluation of Immunohistochemical Staining

Two pathologists independently scored the tissue microarray. In the event of disagreement, they reached a consensus via joint re-evaluation of the tissue microarray using a multihead microscope. At least ten high-power fields were randomly chosen, and at least 500 cells/field were counted in each section. NLK and Ki-67 indices were determined as the percentage of all immunostained cells. The percentage of NLK-positive tumor cells ranged from 1.85% to 81.18%. The mean percentage of positive cells was 19.36%. The samples were considered NLK-positive when the percentage of positive cells was >19.36% and negative when the percentage was ≤19.36%. The immunostaining intensity was graded using three categories: strong (3), moderate (2), weak (1), or negative (0); a semiquantitative score was assigned using the following scale: less than 5% of NLK-positive cells (0), 5%–25% of positive cells (1), 26%–50% of positive cells (2), 50%–75% of positive cells (3), and greater than 75% of positive cells (4). Finally, all these scores were combined. If less than 5% of cells were immunostained, the case was considered negative. Using this procedure, the c-Myb immunohistochemical scores ranged from 0 to 12. Samples were considered positive for c-Myb expression if the score was >3 and negative if the score was ≤3. The scores of core tumor tissue replicates were combined and used as one case. Immunohistochemical evaluation for estrogen receptor (ER), progesterone receptor (PR) and HER-2 was performed by the Department of Pathology, Nantong University Cancer Hospital.

### Cells and Reagents

One commercial normal human breast epithelial cell line (HBL-100) and two human breast cancer cell lines (MDAMB-231 (ER–) and MCF-7 (ER+), purchased from Cancer Hospital of Fudan University, were used in this study. All cell lines were maintained in RPMI 1640 (GibCo BRL, Grand Island, NY, USA) supplemented with 10% heat-inactivated fetal calf serum, 2 mM L-glutamine, and 100 U/mL penicillin-streptomycin mixture (GibCo BRL, Grand Island, NY, USA) at 37°C and 5% CO2.

### MTT Assay and Apoptosis Analysis

Ninety-six-well plates were seeded with 3×10^3^ cells per well. Following overnight incubation, the cells were transfected with pcDNA3.1-EGFP-NLK and pcDNA3.1-EGFP and incubated for 2 days. Then, the medium was removed, and the cells were washed once with PBS. Cell numbers were measured colorimetrically using the Cell Counting Kit (Dojindo, Kumamoto, Japan) by ImmunoMini NJ-2300 (NJ InterMed, Tokyo, Japan) at a test wavelength of 490 nm. Apoptosis was quantified by dual-color flow cytometry analysis after cells were stained with Annexin V-FITC and propidium iodide following the manufacturer’s instructions (Santa Cruz Biotechnology, Santa Cruz, CA). Analysis was performed on a FACScan flow cytometer (BD Biosciences, San Jose, Calif) equipped with CellQuest software. For each sample, 10000 events were collected.

### Western Blot Analysis

Prior to immunoblotting, the cells were washed three times with ice-cold PBS and resuspended in 2× lysis buffer (50 mM Tris–HCl, 120 mM NaCl, 0.5% Nonidet P-40, 100 mM NaF, 200 µM Na3VO4, and protease inhibitor mixture); the frozen tissues were then homogenized in lysis buffer (1% NP-40, 50 mmol/L Tris, pH 7.5, 5 mmol/L EDTA, 1% SDS, 1% sodium deoxycholate, 1% Triton X-100, 1 mmol/L PMSF, 10 mg/mL aprotinin, and 1 mg/mL leupeptin) and incubated for 20 min at 4°C with rocking. The lysates were cleared by centrifugation (10 min, 12,000 rpm, 4°C). Fifty micrograms of total protein was resolved by SDS-PAGE and transferred onto polyvinylidene difluoride membranes (Immbilon, Millipore). The membranes were first blocked with 5% nonfat dry milk and then incubated with anti-NLK (1∶500; sc-28884 Santa Cruz Biotechnology), anti-β-actin (1∶1000; Sigma), anti-c-Myb (1∶500; Santa Cruz Biotechnology), anti-PCNA (1∶1000, Santa Cruz Biotechnology), anti-active caspase3 (1∶1000, Santa Cruz Biotechnology), anti-c-myc (1∶1000, Santa Cruz Biotechnology), and anti-bcl-2 (1∶1000, Santa Cruz Biotechnology). After three washes, the filters were incubated with horseradish peroxidase-conjugated secondary human anti-mouse or anti-rabbit antibodies (1∶1,000; Pierce) for 1 hour at room temperature according to the manufacturer’s instructions. The proteins of interest were detected with an enhanced chemiluminescence system (NEN Life Science Products, Boston, MA).

### Expression Plasmid and Transient Transfection

The cDNA for full-length NLK was a kind gift from Professor Keke Huo (Fudan University). The accession number of NLK is BC064663, and the human NLK-specific primers used were as follows: 5′-CCAGTGACTTTGAGCCTGTC-3′ for the 5′ end and 5′-GATGGCTGAGCAACAGTGG-3′ for the 3′ end. The PCR fragment was cloned into the pcDNA3.1-EGFP expression vector using the EcoRI and XhoI restriction sites. Proper insertion of the clone was confirmed by DNA sequencing, and the plasmid for transfection was prepared using an EndoFree Plasmid Maxi Kit (Qiagen, Tokyo, Japan). Transfection was performed using the Lipofectamine™ 2000 transfection reagent (Invitrogen) according to the manufacturer’s protocol with minor modifications. MCF-7 cells were seeded at a density of 2×10^5^ cells/ml in a 6-well plate 12 hours before transfection. Next, 500 µl of the transfection mixture containing 8 µg of DNA and 20 µl of Lipofectamine 2000 in 450 µl of Opti-MEM (Invitrogen) was added to each well. The cells were harvested 48 hours after transfection and used for the experiment, which was repeated at least three times.

### Proteasome Inhibition Assays

MCF-7 cells were transfected with pcDNA3.1-EGFP-NLK as described above. Thirty-two hours after infection, MG132 (20 µM/L) and DMSO (control) were added to the medium for 16 hours. Forty-eight hours after transfection, whole cell lysates were prepared, and the proteins were analyzed by immunoblotting.

### Statistical Analysis

Statistical significance of the means was determined using Student’s t test. The nonparametric Spearman’s rank correlation coefficient was used to determine the statistical dependence between the expression of NLK, c-Myb and Ki-67. The χ^2^ and Fisher’s exact tests were used to compare the expression of all proteins as groups (positive vs. negative) with various clinicopathological parameters. A P value <0.05 was required for statistical significance. All computations were performed using the SPSS 15.0 software.

## Results

### NLK Expression is Inversely Correlated with c-Myb Expression in Breast Cancer

**Figure 1:**
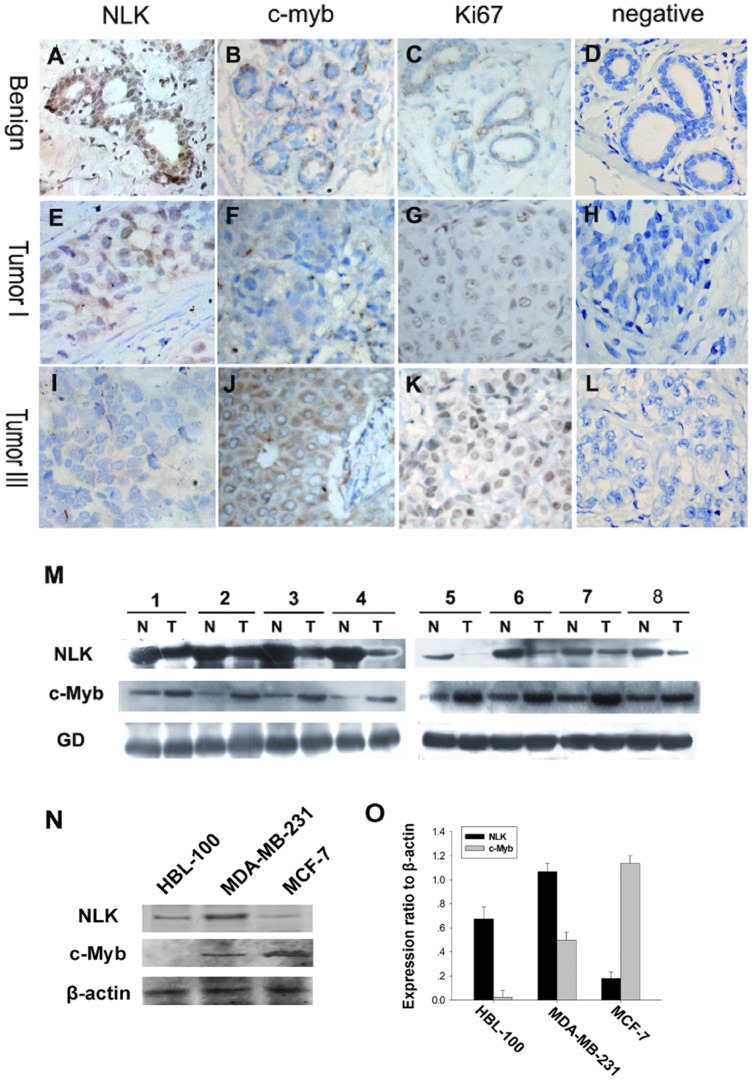
Expression of NLK and c-Myb in human breast cancer. Paraffin-embedded tissue sections were stained with antibodies for NLK, c-Myb and Ki-67 and then counterstained with hematoxylin. Fig. A–C, E–G High NLK expression was observed in benign breast disease and breast carcinoma specimens (grade I), whereas c-Myb and Ki67 levels were low in the same specimens (SP×400). Fig. I-K High levels of c-Myb and Ki67 were observed in grade III tumor cells. In contrast, NLK expression was low. Fig. 1D, H, and L show negative controls for the benign breast disease and the breast carcinoma specimens. Experimental details are described in the Materials and Methods section. (M) Expression of NLK and c-Myb in eight representative paired samples of breast carcinomas and adjacent normal tissues. (N) Western blot analysis of endogenous NLK and c-Myb in a normal human breast epithelial cell line (HBL-100) and two human breast cancer cell lines (MDA-MB-231 (ER–) and MCF-7 (ER+)). β-actin was used as a loading control. The experiment was repeated at least three times. (O) Quantification indicated that MDA-MB-231 cells displayed the highest levels of NLK and the lowest levels of c-Myb among the two tumor cell lines. In contrast, the lowest NLK and highest c-Myb expression were observed in MCF-7 cells.

**Figure 2:**
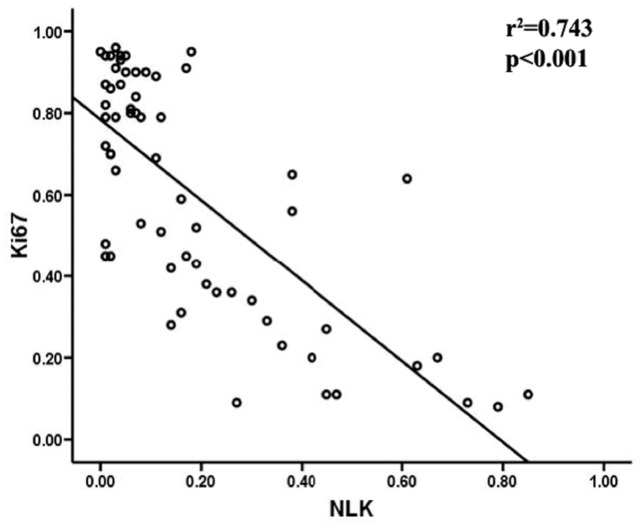
Graphic representation of the relationship between NLK and Ki-67 expression in breast carcinoma. Scatterplot of NLK versus Ki-67 levels with a regression line showing the correlation between the two levels using the Spearman’s correlation coefficient (*P<*0.05).

To determine whether there was a physiological or pathological relationship between the expression of NLK, c-Myb and the proliferation index Ki-67 in breast carcinoma specimens, we examined the levels of NLK, c-Myb and Ki-67 using immunohistochemical staining. Representative examples of NLK, c-Myb and Ki-67 staining are shown in Fig. 1A–I. In most specimens, the proportion of NLK-positive tumor cells was negatively correlated with the proportion of c-Myb-positive and Ki-67-positive tumor cells (Figure 2). The results of these experiments indicated that mammary epithelial and myoepithelial cells from benign breast disease samples showed strong immunoreactivity for NLK in the nucleus (Fig. 1A), with weak to no c-Myb staining (Fig. 1B). Then, we investigated whether there was a correlation between NLK and c-MYB expression and found that high NLK levels (Fig. 1E) were correlated with low c-Myb levels (Fig. 1F) in the same breast carcinoma specimen (grade I); furthermore, low NLK levels (Fig. 1I) were correlated with high c-Myb levels (Fig. 1J) in the same breast carcinoma specimen (grade III). NLK was mainly expressed in the nuclei, while c-Myb was expressed both in the cytoplasm and nuclei of breast cancer cells. The percentage of NLK-positive tumor cells ranged from 1.85% to 81.18%. The mean percentage of positive cells was 19.36%. NLK expression was considered positive when the percentage of positive cells was >19.36% and negative when it was ≤19.36%. NLK immunoreactivity was observed in 42 (67.7%) of the breast cancer cases, whereas there was no detectable NLK immunoreactivity in 20 cases (32.3%). c-Myb expression was considered positive when the score was >3 and negative when it was ≤3. c-Myb immunoreactivity was observed in 58 (93.5%) of the breast cancer cases, whereas there was no detectable c-Myb immunoreactivity in 4 cases (6.5%). The immunohistochemical results of the 62 breast carcinoma specimens are summarized in Table 1. NLK expression was significantly correlated with histological grade and Her2 and Ki67 expression (P<0.05; Table 1). To investigate the cellular proliferation rates of malignant breast tumors, we assessed Ki-67 protein expression in all cases. The results are shown in Fig. 1C, G, and K. Ki-67 expression was lower in benign tumors than in malignant ones (P<0.05). In malignant tumors, the staining intensity was higher in TNM stage III than in stage I (P<0.05). In Fig. 1D, H, and L, negative controls for the benign breast disease and the breast carcinoma specimens are shown. Furthermore, we investigated the abundance of NLK and c-Myb in eight tumors relative to the adjacent normal tissues (Fig. 1M) and found that NLK expression was dramatically decreased in seven of the eight tumors compared to the adjacent normal tissues. However, the same tumor samples had higher expression levels of c-Myb than the paracancerous tissues. To explore the relationship between the two molecules in human breast carcinoma progression and further characterize the relationship between NLK and c-Myb, we investigated their abundance in breast cancer cells by Western blot analysis. First, we examined the basal abundance of NLK and c-Myb in one normal human breast epithelial cell line (HBL-100) and two human breast cancer cell lines (MDA-MB-231 (ER-) and MCF-7 (ER+)). Different expression levels of NLK and c-Myb were observed in all of the cell lines (Fig. 1N, O). As we expected, the relative abundances of NLK and c-Myb appeared to be inversely correlated in the normal human breast epithelial and the two cancer cell lines. MCF-7 cell lines displayed the lowest abundance of NLK and the highest expression level of c-Myb among the three cell lines.

### The Clinical Relevance of NLK and c-Myb in Breast Carcinoma

The frequencies of NLK and c-Myb immunostaining in relation to different clinicopathological parameters are shown in Table 1. For statistical analysis of NLK and c-Myb expression, the carcinoma specimens were divided into two groups (high expressers and low expressers), according to the staining intensity and the percentages of NLK- and c-Myb-positive cells. NLK expression was significantly correlated with histological grade (P<0.001) and Her2 (P = 0.029) and Ki67 (P<0.001) positivity; however, there was no correlation with other prognostic factors, such as age, tumor metastasis, tumor size, histology, and ER or PR status. c-Myb expression was significantly correlated with Ki67 positivity (P<0.001), but no significant correlation was found with other clinicopathologic variables (Table 1). NLK expression was negatively correlated with Ki-67 expression (Spearman’s r = −0.743, P<0.001; Fig. 2) in all of the analyzed breast cancer cases.

### NLK and c-Myb Expression is Cell Cycle-dependent in the MCF-7 Breast Cancer Cell Line

**Figure 3:**
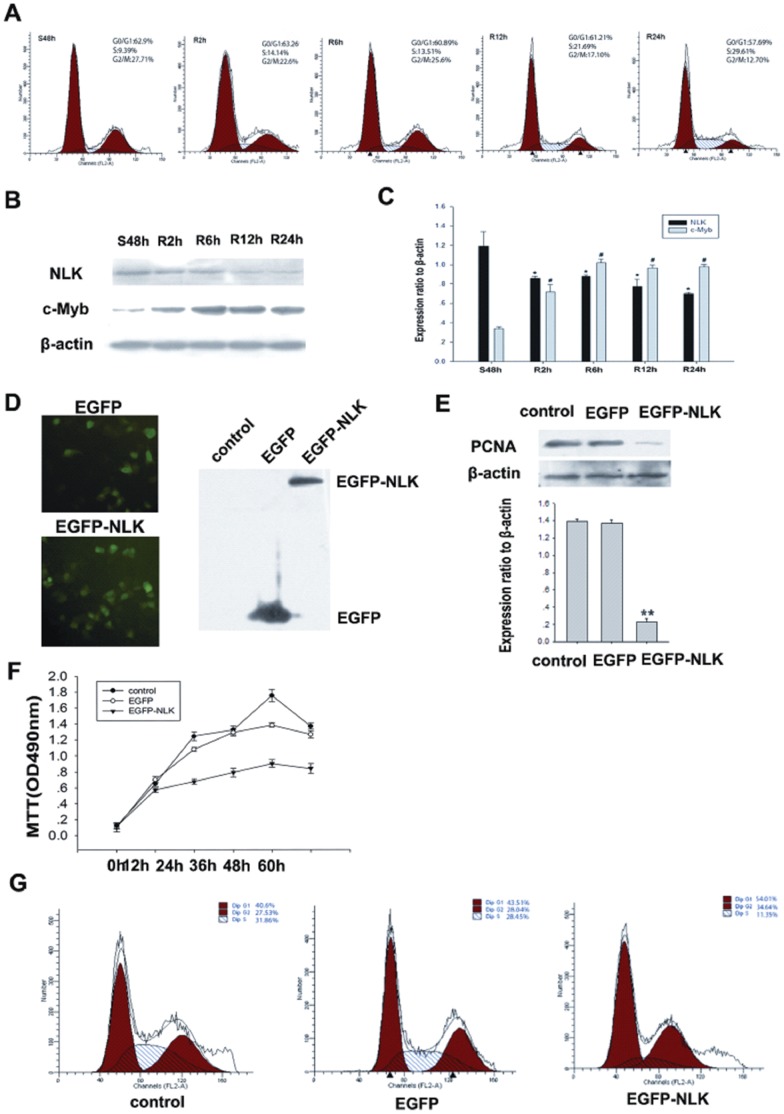
NLK plays an antiproliferative role in breast cancer cells. (A) Flow cytometry analysis of cell cycle progression in MCF-7 cells. Cells that were synchronized at G1 progressed into the cell cycle 0, 2, 6, 12, and 24 hours after serum stimulation. Finally, most of the cells entered S phase. (B) MCF-7 cells were serum starved for 48 hours (S48h); upon serum stimulation, cell lysates were prepared and analyzed by Western blotting using antibodies against NLK, c-Myb and β-actin. β-actin was used as a control for loading and protein integrity. (C) The bar graph demonstrates the ratio of NLK and c-Myb proteins to β-actin at each time point, as determined by densitometry. The data are represented as the mean ± SEM (n = 3, *, #, P<0.01, compared with control: S48h). S: serum starvation; R: serum stimulation. (D). Light microscopy showing that pcDNA3.1-EGFP and pcDNA3.1-EGFP-NLK were expressed in MCF-7 cells. Whole cell extracts were prepared 2 days after the transfection. Proteins were analyzed by immunoblotting using an anti-EGFP antibody. (E). MCF-7 cells were transfected with pcDNA3.1-EGFP-NLK or nothing (control). Western blotting was performed using anti-PCNA and anti-β-actin antibodies. The data are presented as the mean±standard error of three experiments. (F). MCF-7 cells were transfected with pcDNA3.1-EGFP, pcDNA3.1-EGFP-NLK or nothing (control). Cell growth of the transfected cells was assessed by the MTT cell viability assay. Four hours after transfection, cells were maintained in complete media, and cell growth was determined by MTT assay at each indicated time point. The data are presented as the mean±standard error of three experiments. (G). Cell cycle analysis was performed by staining NLK overexpressing MCF-7 cells with PI. MCF-7 cells were transfected with pcDNA3.1-EGFP, pcDNA3.1-EGFP-NLK, or nothing (control). Forty-eight hours after transfection, cells were trypsinized, fixed in 70% alcohol, and incubated for 30 minutes in PBS containing 10 mg/ml of RNase A at 37°C. After the incubation, cells were stained with 5 mg/ml PI.

To investigate the kinetics of NLK expression during the cell cycle, we analyzed cell cycle progression after serum starvation and re-feeding with serum. MCF-7 cells were arrested in the G1 phase by serum deprivation for 48 hours (Fig. 3A). After serum addition, the cells were released from the G1 phase, and the percentage of cells in the S phase increased from 9.39% to 29.61%. In MCF-7 cells, NLK expression increased after serum deprivation for 48 hours and began to decrease 2 hours after serum stimulation, whereas c-Myb expression decreased after serum deprivation for 48 hours and began to increase 2 hours after serum stimulation (Fig. 3B, C). NLK levels were high when there was a greater percentage of cells in G1, whereas c-Myb levels decreased in the G0 and early G1 phases and increased in the S phase. These results provide insights into the kinetics of NLK and c-Myb expression during cell cycle progression.

### NLK Inhibits Proliferation in Breast Cancer Cell Lines

NLK contributes to tumor cell growth through activation of the cell cycle transition in human hepatocellular carcinomas [3]; however, whether this protein kinase has a function in breast cancer cell proliferation remains unclear. Protein expression from the pcDNA3.1-EGFP and pcDNA3.1-EGFP-NLK vectors in MCF-7 cells was confirmed by light microscopy. Whole cell extracts were prepared 2 days after transfection. Protein expression was analyzed by immunoblotting using an anti-EGFP antibody (Fig. 3D), and an anti-PCNA antibody was used to determine proliferative activity. We investigated the effects of NLK on PCNA (a cell proliferation marker) protein levels in MCF-7 cells by Western blotting. As shown in Figure 3E, pcDNA3.1-EGFP had no effect on PCNA expression, while pcDNA3.1-EGFP-NLK significantly reduced PCNA protein levels (p<0.01). To determine the biological effects of NLK overexpression, the growth rate of MCF-7 cells was determined using the MTT assay. As shown in Figure 3F, NLK overexpression significantly decreased the cell growth rate compared to the vector control. Flow cytometric analysis suggested that the expression of pcDNA3.1-EGFP-NLK but not pcDNA3.1-EGFP decreased the percentage of cells in S phase (Fig. 3G). These results suggest that NLK exerts an antiproliferative effect on MCF-7 breast cancer cells.

### NLK Promotes Apoptosis in Breast Cancer Cell Lines

**Figure 4:**
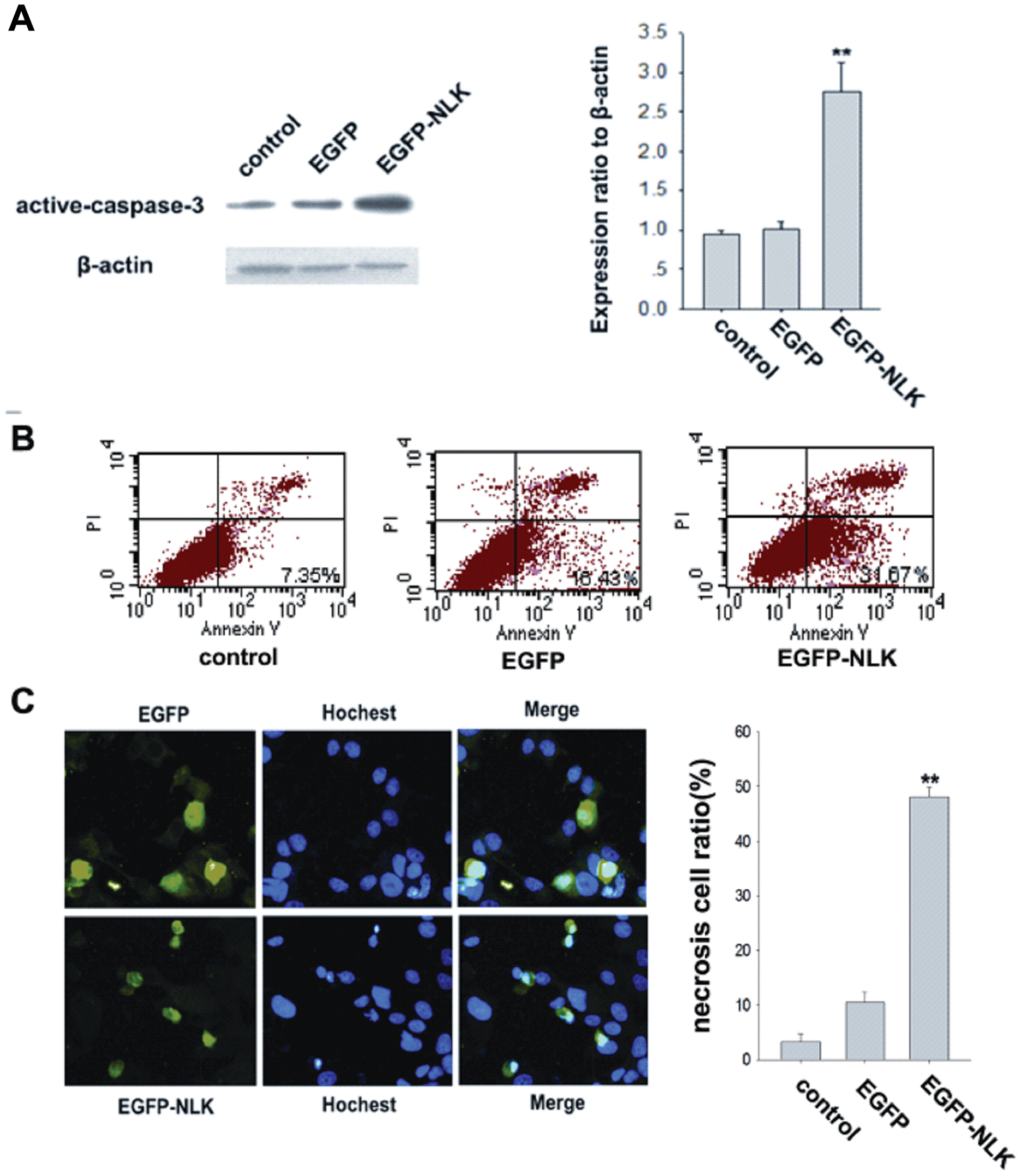
Effect of NLK overexpression on apoptosis of MCF-7 cells. (A). MCF-7 cells were transfected with pcDNA3.1-EGFP, pcDNA3.1-EGFP-NLK or nothing (control). Western blotting was performed using anti-active-caspase-3 and anti-β-actin antibodies. The data are presented as the mean±standard error of three experiments. (B). The effect of NLK overexpression on apoptosis was assessed by Annexin V and PI staining; cells that were double stained with Annexin V and PI were considered to be apoptotic (lower right). Flow cytometry confirmed the induction of apoptosis in MCF-7 cells, for which overexpression of NLK caused an increase in apoptosis. (C). MCF-7 cells were transfected with pcDNA3.1-EGFP, pcDNA3.1-EGFP-NLK or nothing (control). Forty-eight hours after transfection, DAPI-stained nuclei were visualized by fluorescence microscopy, and images were taken. The bar chart shows the ratio of necrotic to transfected cells. The data are represented as the mean ± SEM (n = 3, *, #, P<0.01, compared with control).

NLK has been shown to play a role in the induction of apoptosis in prostate cancer cells [10]; however, whether this protein kinase has a function in breast cancer cell apoptosis remains unclear. Using Western blotting, we examined the expression of active caspase-3, which is a known marker of apoptosis. As shown in Figure 4A, pcDNA3.1-EGFP had no effect on active caspase-3 expression, whereas pcDNA3.1-EGFP-NLK significantly upregulated the levels of active caspase-3 protein (p<0.01). To assess cellular apoptosis, transfected cells were stained with Annexin V and analyzed by flow cytometry. As shown in Figure 4B, flow cytometry analysis following PI and Annexin V staining indicated that cellular apoptosis (upper right quadrant in the dot plot graphs) was affected by NLK overexpression. Nuclear condensation and fragmentation, typical characteristics of apoptotic cells, were observed in numerous floating cells stained with DAPI after 2 days of wild-type NLK expression in MCF-7 cells (Fig. 4C). These results suggest that NLK induces apoptosis in MCF-7 cells.

### NLK Downregulated c-myc and bcl-2 by Degrading c-Myb in Human Breast Carcinoma

**Figure 5:**
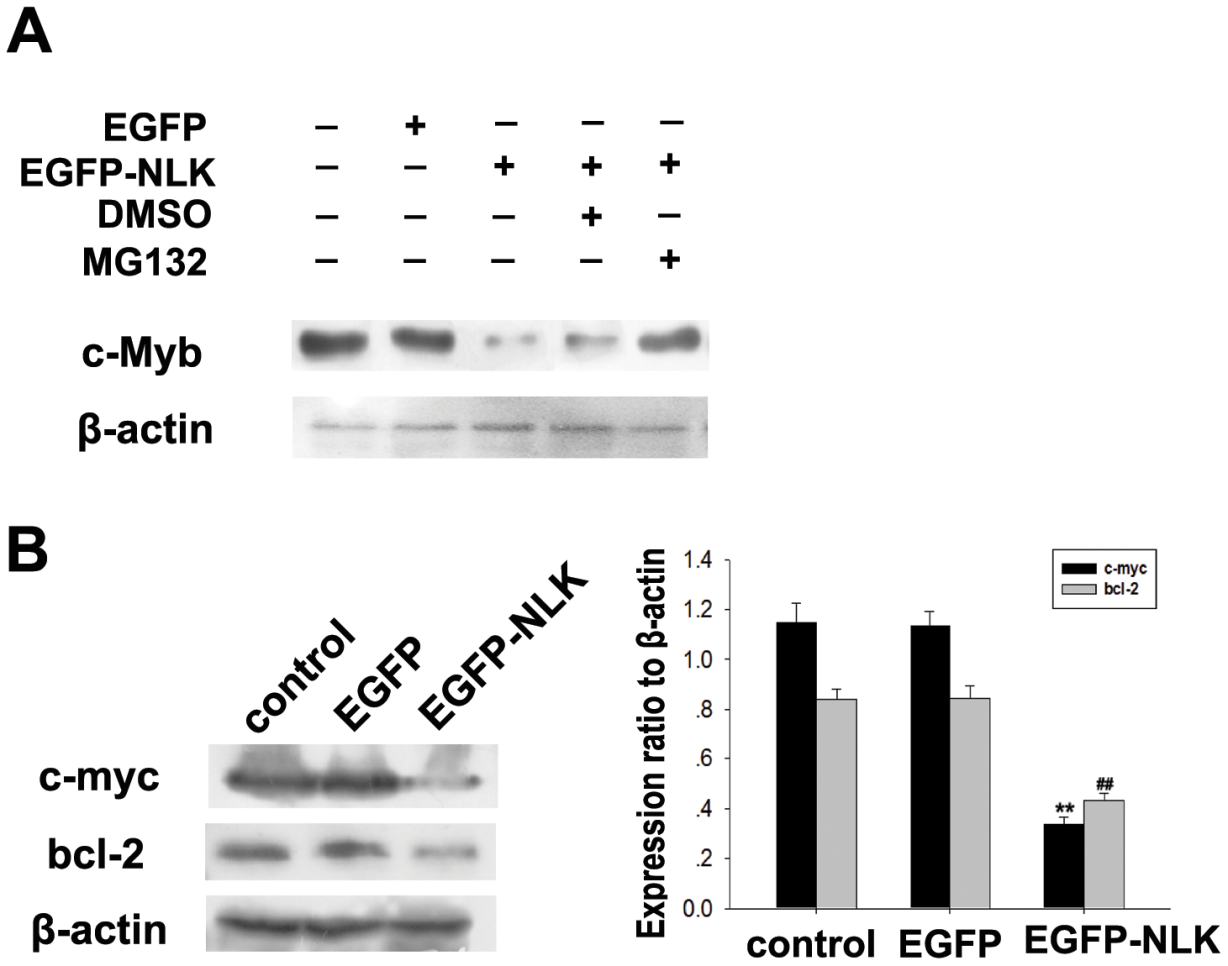
NLK induces c-Myb degradation in MCF-7 cells. (A). MCF-7 cells were transfected with the pcDNA3.1-Myc-NLK or pcDNA3.1-Myc expression vectors, and 32 hours posttransfection, 26S proteasome inhibitor MG132 and DMSO as control were added for 16 hours. At 48 hours posttransfection, whole cell lysates were prepared, and proteins were analyzed by immunoblotting. The data were representative of three experiments. (B). pcDNA3.1-Myc-NLK or empty vector was transfected into MCF-7 cells using Lipofectamine 2000. Western blotting was performed using antibodies against c-myc, Bcl-2 and β-actin. The bar chart demonstrates the ratio of c-myc and Bcl-2 proteins to β-actin as determined by densitometry. The data are represented as the mean ± SEM (n = 3, *, #, P<0.01, compared with control).

Chie Kanei-Ishii found that c-Myb protein is phosphorylated and degraded by the Wnt-1 signaling pathway involving TAK1, HIPK2, and NLK [2]. HIPK2 and NLK directly bind to c-Myb, and NLK phosphorylates c-Myb at multiple sites, resulting in its ubiquitination and proteasome-dependent degradation [32]. To investigate whether NLK overexpression would downregulate c-Myb levels in breast carcinoma cells, we transfected MCF-7 cells with pcDNA3.1-EGFP-NLK or an empty vector and measured NLK and c-Myb levels 48 hours after transfection. As shown in Fig. 5A, NLK overexpression led to a significant decrease in c-Myb levels compared to the vector control. Furthermore, the downregulation of c-Myb by NLK overexpression could be inhibited in cells that had been treated with the 26S proteasome inhibitor MG132 but not in those treated with DMSO (Fig. 5A). This finding indicated that NLK promoted c-Myb degradation through proteolysis and that this process was sensitive to 26S proteasome inhibitors. These data provide direct evidence for a functional relationship between NLK and c-Myb in breast carcinoma cells. It is already known that c-Myb regulates the levels of Bcl-2 and c-myc, which are modulators of apoptosis and cell cycle arrest, respectively [33], [34]. We examined the levels of Bcl-2 and c-myc by Western blotting and found that ectopic expression of NLK downregulated c-myc, which is a target gene of c-Myb (Fig. 5B).

## Discussion

It is known that most breast carcinomas occur as a result of alterations in oncogenes and tumor suppressor genes, which regulate the signal transduction pathways involved in cell proliferation, differentiation and apoptosis. In the past decade, a number of transcription factors and cofactors have been demonstrated to be phosphorylated and regulated by NLK [35]. Aberrant expression of NLK was correlated with proliferation and apoptosis in hepatocellular carcinoma [3], prostate cancer [10] and colon cancer [36]. The overexpression of c-Myb has been associated with oncogenic activity and poor prognosis in several human cancers, including T cell leukemia, acute myelogenous leukemia, colorectal tumors, and, most recently, adenoid cystic carcinomas [22], [37]. c-Myb is a potential tumor suppressor in luminal breast cancer [38].

In this study, we used immunohistochemical analysis to assess NLK expression in breast carcinoma cases. We found an inverse correlation between the expression of NLK and that of Ki-67, which is a marker of cell proliferation that is specifically expressed in the nuclei of cells in late G1 to S phase. These findings suggested that alterations in NLK protein expression could affect tumor development. Reduced NLK expression was closely associated with the breast cancer malignancy. Malignant transformation is a complex process that may be regulated, at least in part, by reduced expression of NLK. We found a statistically significant correlation between the expression of NLK and c-Myb. Furthermore, we observed an inverse correlation between NLK and c-Myb expression in breast carcinomas, with low NLK expression being a poor prognostic factor. The negative correlation between NLK and c-Myb expression was also observed in normal human breast epithelial and breast cancer cell lines, regardless of estrogen receptor expression status. Taken together, our observations supported the hypothesis that NLK might contribute to the progression of breast carcinoma as a regulator of c-Myb.

In our study, the cause of growth suppression in the breast cells by wild-type NLK could be either cell cycle arrest, increased apoptosis rate or both. The overexpression of NLK resulted in suppressed cell growth (Fig. 3F) and an arrest in the cell cycle transition (Fig. 3G). These findings suggest that overexpression of NLK might act as an inhibitor of breast cancer cell proliferation by blocking cell growth. Previous studies have suggested that NLK overexpression induced apoptosis in human colon cancer cells. Our flow cytometry data, as well as the nuclear condensation and fragmentation phenotype observed using microscopy, indicated that NLK expression increased the number of apoptotic cells. The present study showed that induction of wild-type NLK in the human breast carcinoma cell line MCF-7 caused cell growth suppression and apoptosis induction.

c-myc is a target of c-Myb, and activation of the c-myc gene is required for Myb-mediated transformation [33]. Bcl-2 is a direct target gene of the protooncogene c-Myb [34]. Several studies have shown that c-Myb regulates bcl-2 gene expression in hematopoietic cells [39], [40]. Bcl-2 expression is reduced and apoptosis is increased in the colonic epithelium of embryos with a disrupted c-myb gene [41]. In our study, c-Myb expression was induced from late G1 to S phase, whereas NLK was decreased simultaneously with the accumulation of c-Myb (Fig. 3B,C). We also found that c-Myb expression was significantly decreased by NLK overexpression, resulting in lower levels of c-myc and bcl-2 in MCF-7 cells (Fig. 5A). We hypothesize that NLK might be involved in the degradation of c-Myb and that its target genes c-myc and bcl-2 might be potential prognostic factors for breast carcinoma. The underlying mechanism by which NLK regulates c-Myb expression remains to be elucidated; however, NLK’s role in human breast cancer development and progression by attenuating c-Myb activity makes it an attractive target for cancer therapy.

Other than the NLK/c-Myb pathway, additional mitogenic cascades have been found to be associated with NLK function in cancer. Jun Yasuda proposed that NLK might function as a tumor suppressor in colon cancer by inhibiting the Wnt/β-catenin pathway, which suppresses the transcriptional activity of the β-catenin/T-cell factor (TCF) complex by phosphorylating TCF. More studies are needed to address the possibility of other pathways regulating NLK function in breast cancer cells, which has been reported previously for other cell lines.

This study demonstrates the consequences of NLK dysregulation in human breast carcinoma. The overexpression of NLK suppressed tumor cell proliferation and transforming potential via the transcriptional inhibition of c-Myb. This is the first study showing that NLK is dysregulated in human breast carcinoma and suggesting that NLK might contribute to tumor cell growth and apoptosis. The data suggest that NLK might be associated with the development of aggressiveness in breast carcinoma. We hypothesize that the functional interaction between NLK and c-Myb has a critical role in breast carcinoma progression. Agents that stabilize NLK or enhance the NLK/c-Myb interaction may be of great value in the treatment of breast carcinoma. Further studies will focus on the molecular mechanisms of NLK-induced tumor development, as well as on developing strategies to upregulate the protein or modulate its function for potential therapeutic applications. This study’s aim was to further our understanding of NLK’s role in breast cancer. In conclusion, NLK plays an antineoplastic role in breast cancer via regulating its target gene c-Myb.

## References

[1] Sonia M, Hailong W, Priyasri C, Kounosuke W (2007) Wnt pathway and breast cancer. Frontiers in Bioscience 12: 4020–33. 17485355 10.2741/2368

[2] Chie KI, Jun NT, Jun T, Teruaki N, Tohru I, et al (2004) Wnt-1 signal induces phosphorylation and degradation of c-Myb protein via TAK1, HIPK2, and NLK. Genes Dev 18: 816–29. 15082531 10.1101/gad.1170604 PMC387421

[3] Ishikawa T, Shimizu D, Kito A, Ota I, Sasaki T, et al (2012) Breast cancer manifested by hematologic disorders. J Thorac Dis 4: 650–4. 23205295 10.3978/j.issn.2072-1439.2012.10.17 PMC3506789

[4] Eriko O, Toshiyasu G, Atsushi S, Misun K, Shunichiro I, et al (2010) Nemo-Like Kinase, an Essential Effector of Anterior Formation,Functions Downstream of p38 Mitogen-Activated Protein Kinase. Mol Cell Biol 30: 675–83. 19933839 10.1128/MCB.00576-09 PMC2812222

[5] Daemen A (2012) An update on the genomic landscape of breast cancer: new opportunity for personalized therapy? Transl Cancer Res 1: 279–82.

[6] Barbara KB, Benjamin AP, Raymond LE (1998) Nlk is a murine protein kinase related to Erk/MAP kinases and localized in the nucleus. Proc. Natl. Acad. Sci. U. S. A. 95: 963–8. 10.1073/pnas.95.3.963 PMC18639 9448268

[7] Ivana M, Kristi C, Sharon MG, Kristen M, Esther MV (2002) Drosophila nemo is an essential gene involved in the regulation of programmed cell death. Mech Dev 119: 9–20. 12385750 10.1016/s0925-4773(02)00289-7

[8] Kaletta T, Schnabel H, Schnabel R (1997) Binary specification of the embryonic lineage in Caenorhabditis elegans. Nature 390: 294–8. 9384382 10.1038/36869

[9] Verheyen EM, Mirkovic I, MacLean SJ, Langmann C, Andrews BC, et al (2001) The tissue polarity gene nemo carries out multiple roles in patterning during Drosophila development. Mech Dev 101: 119–32. 11231065 10.1016/s0925-4773(00)00574-8

[10] Katayoon HE, Lisha GB, Tiffany EMP, Xizhang S, Robert LV, et al (2009) Nemo-LikeKinase Induces Apoptosis and Inhibits Androgen Receptor Signaling in Prostate Cancer Cells. Prostate 69: 1481–92. 19514049 10.1002/pros.20998 PMC2908180

[11] Cui G, Li Z, Shao B, Zhou WF, Liu T, et al (2011) Clinical and biological significance of Nemo-like kinase expression in glioma. J Clin neurosci 18: 271–5. 21177110 10.1016/j.jocn.2010.05.037

[12] Graf T, Altieri M, Venturelli D (1992) Myb: a transcriptional activator linking proliferation and differentiation in hematopoietic cells. Curr Opin Genet Dev 2: 249–55. 1638119 10.1016/s0959-437x(05)80281-3

[13] Weston K (1998) Myb proteins in life, death and differentiation. Curr Opin Genet Dev 8: 76–81. 9529609 10.1016/s0959-437x(98)80065-8

[14] Gewirtz AM, Calabretta B (1988) A c-myb antisense oligodeoxynucleotide inhibits normal human hematopoiesis in vitro. Science 242: 1303–6. 2461588 10.1126/science.2461588

[15] Gewirtz AM, Anfossi G, Venturelli D, Valpreda S, Sims R, et al (1989) G1/S transition in normal human T-lymphocytes requires the nuclear protein encoded by c-myb. Science 245: 180–3. 2665077 10.1126/science.2665077

[16] Anfossi G, Gewirtz AM, Calabretta B (1989) An oligomer complementary to c-myb-encoded mRNA inhibits proliferation of human myeloid leukemia cell lines. Proc Natl Acad Sci U S A 86: 3379–83. 2541445 10.1073/pnas.86.9.3379 PMC287136

[17] Melani C, Rivoltini L, Parmiani G, Calabretta B, Colombo MP (1991) Inhibition of proliferation by c-myb antisense oligodeoxynucleotides in colon adenocarcinoma cell lines that express c-myb. Cancer Res 51: 2897–901. 2032228

[18] Zorbas M, Sicurella C, Bertoncello I, venter D, Ellis S, et al (1999) c-Myb is critical for murine colon development. Oncogene 18: 5821–30. 10523863 10.1038/sj.onc.1202971

[19] Hodges LC, Cook JD, Lobenhofer EK, Li L, Bennett L, et al (2003) Tamoxifen functions as a molecular agonist inducing cell cycle-associated genes in breast cancer cells. Mol Cancer Res 1: 300–11. 12612058

[20] Kauraniemi P, Hedenfalk I, Persson K, Duggan DJ, Tanner M, et al (2000) MYB oncogene amplification in hereditary BRCA1 breast cancer. Cancer Res 60: 5323–8. 11034064

[21] Villa AE, Guzman LA, Poptic EJ, Labhasetwar AD, Souza S, et al (1995) Effects of antisense c-myb oligonucleotides on vascular smooth muscle cell proliferation and response to vessel wall injury. Circ Res 76: 505–13. 7895327 10.1161/01.res.76.4.505

[22] Annamaria B, Barbara B, Igea D (2001) c-Myc and Bcl-x over-expression predicts poor prognosis in colorectal cancer: clinical and experimental findings. Am J Pathol 158: 1289–99. 11290547 10.1016/S0002-9440(10)64080-1 PMC1891926

[23] Thompson M, Flegg R, Westin E, Ramsay R (1997) Microsatellite deletions in the c-myb transcriptional attenuator region associated with over-expression in colon tumour cell lines. Oncogene 14: 1715–23. 9135073 10.1038/sj.onc.1201007

[24] Thompson M, Flegg R, Westin E, Ramsay R (1997) Microsatellite deletions in the c-myb transcriptional attenuator region associated with over-expression in colon tumour cell lines. Oncogene 14: 1715–23. 9135073 10.1038/sj.onc.1201007

[25] Ramsay RG, Thompson MA, Hayman JA, Reid G, Gonda TJ, et al (1992) Myb expression is higher in malignant human colonic carcinoma and premalignant adenomatous polyps than in normal mucosa. Cell Growth Differ 3: 723–30. 1445802

[26] Melani C, Rivoltini L, Parmiani G, Calabretta B, Colombo M (1991) Inhibition of proliferation by c-myb antisense oligodeoxynucleotides in colon adenocarcinoma cell lines that express c-myb. Cancer Research 51: 2897–901. 2032228

[27] Yvette D, Ramsay GR, Thomas JG (2010) MYB suppresses differentiation and apoptosis of human breast cancer cells. Breast Cancer Research 12: R55. 20659323 10.1186/bcr2614 PMC2949644

[28] Toshihiro K, Teruaki N, Chie KI, Yoichi S, Shunsuke I (2005) The Wnt–NLK Signaling Pathway Inhibits A-Myb Activity by Inhibiting the Association with Coactivator CBP and Methylating Histone H3. Mol Bio Cell 16: 4705–13. 16055500 10.1091/mbc.E05-05-0470 PMC1237076

[29] Wodarz A, Nusse R (1998) Mechanisms of Wnt signaling in development. Annu Rev Cell Dev Biol 14: 59–88. 9891778 10.1146/annurev.cellbio.14.1.59

[30] Civenni G, Holbro T, Hynes NE (2003) Wnt1 and Wnt5a induce cyclin D1 expression through ErbB1 transactivation in HC11 mammary epithelial cells. EMBO Rep 4: 166–71. 12612606 10.1038/sj.embor.embor735 PMC1315833

[31] Jordan BK, Shen JH, Olaso R, Ingraham HA, Vilain E (2003) Wnt4 over-expression disrupts normal testicular vasculature and inhibits testosterone synthesis by repressing steroidogenic factor 1/beta-catenin synergy. Proc Natl Acad Sci USA 100: 10866–71. 12949260 10.1073/pnas.1834480100 PMC196894

[32] Chie KI, Teruaki N, Tsuyoshi T, Nobumoto W, Keiichi IN, et al (2008) Fbxw7 Acts as an E3 Ubiquitin Ligase That Targets c-Myb for Nemo-like Kinase (NLK)-induced Degradation. J Biol Chem 283: 30540–8. 18765672 10.1074/jbc.M804340200 PMC2662147

[33] Atul K, Clement ML, Reddy EP (2003) c-Myc Is Essential but Not Sufficient for c-Myb-mediated Block ofGranulocytic Differentiation. J Biol Chem 278: 11480–8. 12525485 10.1074/jbc.M300080200

[34] Feng F, Michael AR, Charles VC (2009) Role of c-Myb during Prolactin-Induced Signal Transducer and Activator of Transcription 5a Signaling in Breast Cancer Cells. Endocrinology 150: 1597–606. 19036881 10.1210/en.2008-1079 PMC2659289

[35] Kim Sh, Kim YS, Lee JW, Chung Jk (2010) Regulation of FOXO1 by TAK1-Nemo-like Kinase Pathway. J Biol Chem 285: 8122–9. 20061393 10.1074/jbc.M110.101824 PMC2832963

[36] Jun Y, Akira T, Tesshi Y, Michiie S, Takao S, et al (2003) Nemo-like kinase induces apoptosis in DLD-1 human colon cancer cells. Biochem Bioph Res Co 308: 227–33. 10.1016/s0006-291x(03)01343-3 12901858

[37] Persson M, Andren Y, Mark J, Horlings HM, Persson F, et al (2009) Recurrent fusion of MYB and NFIB transcription factor genes in carcinomas of the breast and head and neck. Proc Natl Acad Sci 106: 18740–4. 19841262 10.1073/pnas.0909114106 PMC2773970

[38] Aaron RT, Joel SP, Katherine AH, Charles MP (2010) Potential Tumor Suppressor Role for the c-Myb Oncogene in Luminal Breast Cancer. PLoS ONE 5: e13073. 20949095 10.1371/journal.pone.0013073 PMC2951337

[39] Frampton J, Ramqvist T, Graf T (1996) v-Myb of E26 leukemia virus up-regulates bcl-2 and suppresses apoptosis in myeloid cells. Genes Dev 10: 2720–31. 8946913 10.1101/gad.10.21.2720

[40] Salomoni P, Perrotti D, Martinez R, Francheschi C, Calahretta B (1997) Resistance to apoptosis in CTLL-2 cells constitutively expressing c-Myb is associated with induction of BCL-2 expression and Myb-dependent regulation of bcl-2 promoter activity. Proc Nati Acad Sci USA 94: 3296–301. 10.1073/pnas.94.7.3296 PMC20363 9096387

[41] Zorbas M, Sicurella C, Bertoncello I, Venter D, Ellis S, et al (1999) c-Myb is critical for murine colon development. Oncogene 18: 5821–30. 10523863 10.1038/sj.onc.1202971

